# Microbial composition of Kombucha determined using amplicon sequencing and shotgun metagenomics

**DOI:** 10.1111/1750-3841.14992

**Published:** 2020-01-20

**Authors:** Muzaffer Arıkan, Alex L. Mitchell, Robert D. Finn, Filiz Gürel

**Affiliations:** ^1^ Regenerative and Restorative Medicine Research Center Istanbul Medipol Univ. 34810 Istanbul Turkey; ^2^ European Molecular Biology Laboratory European Bioinformatics Inst. (EMBL‐EBI) Wellcome Trust Genome Campus, Hinxton Cambridge United Kingdom; ^3^ Molecular Biology and Genetics Dept. Faculty of Science, Istanbul Univ. 34134 Istanbul Turkey

**Keywords:** Kombucha, shotgun metagenomics, fermented tea, 16S, ITS

## Abstract

Kombucha, a fermented tea generated from the co‐culture of yeasts and bacteria, has gained worldwide popularity in recent years due to its potential benefits to human health. As a result, many studies have attempted to characterize both its biochemical properties and microbial composition. Here, we have applied a combination of whole metagenome sequencing (WMS) and amplicon (16S rRNA and Internal Transcribed Spacer 1 [ITS1]) sequencing to investigate the microbial communities of homemade Kombucha fermentations from day 3 to day 15. We identified the dominant bacterial genus as *Komagataeibacter* and dominant fungal genus as *Zygosaccharomyces* in all samples at all time points. Furthermore, we recovered three near complete *Komagataeibacter* genomes and one *Zygosaccharomyces bailii* genome and then predicted their functional properties. Also, we determined the broad taxonomic and functional profile of plasmids found within the Kombucha microbial communities. Overall, this study provides a detailed description of the taxonomic and functional systems of the Kombucha microbial community. Based on this, we conject that the functional complementarity enables metabolic cross talks between *Komagataeibacter* species and *Z. bailii*, which helps establish the sustained a relatively low diversity ecosystem in Kombucha.

## 1 INTRODUCTION

Kombucha is a fermented drink that was first consumed in China more than 2000 years ago, and has since become popular in many countries (Jayabalan, Malbaša, Lončar, Vitas, & Sathishkumar, 2014). Due to its claimed positive effects on human health, a number of research studies have been conducted on the biochemical characteristics, microbiology, toxicity, cellulose production, and fermentation dynamics of this beverage (Greenwalt, Steinkraus, & Ledford, 2000; Jayabalan et al., 2014; Rosma, Karim, & Bhat, 2012; Sreeramulu, Zhu, & Knol, 2000). Until recently, studies on the microbial ecology of Kombucha have determined the bacterial and fungal diversity through culture‐based methods or sequencing of the phylogenetic marker genes (Chakravorty et al., 2016; Coton et al., 2017; De Filippis, Troise, Vitaglione, & Ercolini, 2018; Marsh, O'Sullivan, Hill, Ross, & Cotter, 2014; Reva et al., 2015).

The development of next‐generation sequencing (NGS) technologies have advanced the metagenomics field by reducing costs and increasing throughput (Ari & Arikan, 2016). To date, several NGS‐based microbiome studies investigating the potential effects of different parameters, such as temperature (De Filippis et al., 2018), tea type (Coton et al., 2017), geography (Marsh et al., 2014), and nutritional sources (Reva et al., 2015), on Kombucha have also been published.

Although the aforementioned amplicon‐based microbiome studies have provided important knowledge about the microbial dynamics of Kombucha, they do not allow functional characterization. Whole metagenome shotgun (WMS) sequencing offers important advantages, such as elimination of PCR bias and recovery of microbial genomes (De Filippis, Parente, & Ercolini, 2017). Moreover, the development of new bioinformatics tools have facilitated the analysis of shotgun sequencing results, and thus contributed to the widespread use of WMS (Oulas et al., 2015).

The aim of this study was to determine microbial composition and functional characteristics of two Turkish Kombucha samples throughout the fermentation process. The harvesting was carried out at days 3, 10, and 15 of the fermentation from both the pellicle and liquid phases. Isolated metagenomic DNA samples were analyzed using WMS. NGS‐based amplicon (16S rRNA gene and Internal Transcribed Spacer 1 [ITS1]) sequencing was also applied to verify the WMS‐based taxonomic analysis results. Detailed taxonomic and functional characteristics of the Kombucha samples were determined through genome assembly and analysis.

## 2 MATERIALS AND METHODS

**Figure 1:**
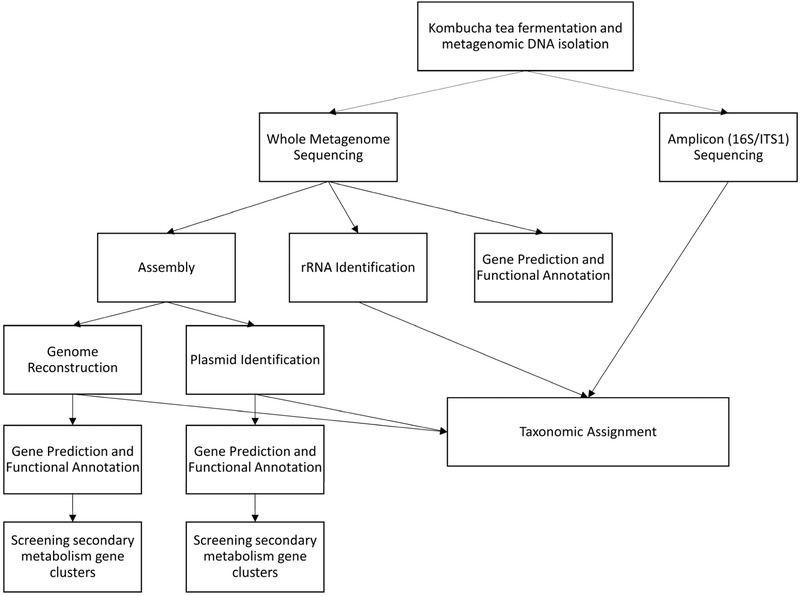
Overall analysis strategy.

The overall analysis strategy of the study is presented in Figure 1.

### 2.1 Kombucha tea fermentation

Two independent Kombucha samples were obtained from local families living in the Adana province of Turkey. Identical fermentation conditions were applied to both samples. For each Kombucha sample, 1,350 mL tap water was boiled, and 150 g sucrose added. Then, 9 g tea leaves were added for 10 min incubation and removed. After cooling the prepared tea to the room temperature, 150 mL from a previously fermented Kombucha tea was added. The total volume for each sample was divided into three 500 mL batches. A triangle shaped cellulose biofilm (pellicle) (approximately 100 mm^2^) was added to each batch. The containers were covered with a thin fabric and secured with a rubber band and batches were incubated at 28 °C. The batches were harvested at different time intervals (3, 10, and 15 days of fermentation).

### 2.2 Metagenomic DNA isolation

Metagenomic DNA was isolated using DNeasy PowerFood Microbial Kit (Qiagen, Hilden, Germany) with modifications to the manufacturer's protocol. In order to isolate metagenomic DNA from the tea phase, 500 mL liquid sample was centrifuged at 6,000 rpm for 15 min and supernatant was discarded. The pellet was washed with 2 mL TE buffer two times, centrifuged at 13,000 rpm for 5 min and the pellet was incubated in 1 mL TES buffer (50 mM Tris, 1 mM EDTA [pH 8], 8.7% sucrose) including 250 u Lyticase (Sigma‐Aldrich, St Louis, MO, USA), 25 u Mutanolysin (Sigma‐Aldrich), and 1,000 u Lysozyme (Sigma‐Aldrich) for 1 hr at 37 °C. The sample was centrifuged at 6,000 rpm for 15 min and supernatant was discarded. The pellet was dissolved in 450 µL PF1 lysis buffer (PowerFood Microbial DNA Isolation Kit) and transferred to a new tube with microbeads. 2% (w/v) Polyvinylpyrrolidone (PVP) (Sigma‐Aldrich) was added to the mix. The mix was incubated at 70 °C for 1 hr and vortexed every 10 min. After this incubation step, the manufacturer's protocol was applied without any modification. For the pellicle, the newly formed pellicle was disrupted and homogenized in 200 mL acetate buffer (1M, pH: 5) using a blender for 30 s. Then, 500 unit cellulase (Sigma‐Aldrich) was added and the homogenized mix was incubated at 40 °C for 1 hr. The sample was centrifuged at 6,000 rpm for 15 min and supernatant was discarded. After this step, the same liquid phase protocol was applied to isolate metagenomic DNA.

### 2.3 Next‐generation sequencing

The bacterial diversity in Kombucha samples was determined by sequencing using F‐5′‐CCTACGGGNGGCWGCAG‐3′ and R‐5′‐GACTACHVGGGTATCTAATCC‐3′ primers (Klindworth et al., 2013) targeting V3‐V4 region of 16S rRNA gene (*E. coli* positions 341–357 and 785–805). ITS1F‐5′‐CTTGGTCATTTAGAGGAAGTAA‐3′ (Gardes & Bruns, 1993) and ITS2R‐5′‐GCTGCGTTCTTCATCGATGC‐3′ (White, Bruns, Lee, & Taylor, 1990) primers were used for the investigation of fungal diversity. PCR was performed using HotStarTaq Plus Master Mix Kit (Qiagen) and PCR products were checked in 2% agarose gel to determine the success of amplification. Barcoded amplicons from different samples were pooled together in equal concentrations and purified using calibrated Ampure XP beads (Agencourt Bioscience Co., Beverly, MA, USA). PCR amplicons were sequenced using the Illumina MiSeq platform (Illumina, San Diego, CA, USA) and TruSeq 2 × 300 bp PE kit (Illumina) according to the manufacturer's instructions.

For WMS sequencing, quality control, fragmentation, hybridization and sequencing steps were applied to the isolated metagenomic DNA using Nextera XT kit (2 × 150 PE) and Illumina HiSeq X Ten platform. Real Time Analysis 2 software was used for raw image generation and base calling. Binary base call files were converted into FASTQ using the Illumina package bcl2fastq v2.15.0. The demultiplexing option (–barcode‐mismatches) was set to default (value: 1).

### 2.4 Bioinformatics analyses

#### 2.4.1 Quality control

MGnify version 4.1 (Mitchell et al., 2018) was used for merging paired end reads and quality control, which includes trimming, length filtering, and ambiguous base filtering by the SeqPrep (https://github.com/jstjohn/SeqPrep) and Trimmomatic (Bolger, Lohse, & Usadel, 2014) tools.

Since the preliminary taxonomic analysis displayed an inconsistency between the small subunit ribosomal ribonucleic acid (SSU rRNA) gene‐based and large subunit ribosomal ribonucleic acid (LSU rRNA) gene‐based results for one WMS sample (BP3), the potential reasons for these differences were investigated. Due to an unusually high number of SSU rRNA gene sequences (36,195,266) compared to LSU rRNA gene sequences (856,622), BP3 was examined for the potential 16S rRNA gene amplicon contamination. To do this, Infernal (Nawrocki & Eddy, 2013) (running in HMM‐only mode) and a library of ribosomal RNA models from Rfam 12.2 (Nawrocki et al., 2015) (families comprising Rfam clans CL00111 and CL00112, representing the SSU and LSU, respectively) were used to generate a coordinate table that included SSU rRNA gene sequences and their start‐end match positions on the 16S rRNA gene template. The coverage distribution for start and end coordinates of reads showed a clear peak at the positions 338‐344 and 644‐704 whereas a uniform distribution would be expected due to random fragmentation of input DNA for shotgun sequencing (S2 File). After the removal of the sequences having start or end coordinates at these peak positions, SSU rRNA gene‐based taxonomic analysis results were in agreement with LSU rRNA gene‐based results, a further confirmation of 16S rRNA gene amplicon contamination. The investigation of all samples showed that four more samples (BS3, BP10, BP15, AS15) had peaks at similar positions. Thus, the WMS experiments were repeated for four samples (BP3, BP10, BP15, BS3), confirming the presence of contaminants in the original sequencing runs. Unfortunately, it was not possible to repeat the sequencing for AS15 due to the lack of available input DNA. Instead, we have applied a decontamination protocol which includes the removal of the reads having start or end coordinates at the determined peak positions (338‐344 and 644‐704) on the 16S rRNA template. And then, remaining SSU rRNA gene sequences were used for taxonomic analysis. To understand potential implications of this decontamination protocol for downstream analysis, we compared SSU rRNA gene‐based taxonomic analysis results of the decontaminated samples BP3, BP10, BP15, and BS3 to the corresponding re‐sequenced version of the same samples, which showed consistency between the decontaminated and re‐sequenced datasets, indicating that the data from AS15 should not significantly impact our results and conclusions (S2 File). Thus, 192,767 sequences in AS15 that were suspected to be contaminants were removed from the raw data and the cleaned data for AS15, composed of 205,987,301 reads, was uploaded to the ENA for further analyses.

#### 2.4.2 Taxonomic analyses

Taxonomic analysis of WMS data was performed using MGnify version 4.1 (http://www.ebi.ac.uk/metagenomics). Briefly, the LSU and SSU rRNA gene sequences in merged and quality checked reads were identified using Infernal and used for taxonomic analysis. Taxonomic assignments were carried out using MapSeq version 1.2 and SILVA v128 database. Both eukaryotic and prokaryotic LSU and SSU rRNA gene fragments are identified in the MGnify pipeline which allows determination of the relative abundances of both domains’ community members thus calculation of bacteria/fungi ratio. In addition, ITSx (Bengtsson‐Palme et al., 2013) was used to extract ITS sequences from WMS data in order to conduct an ITS‐based taxonomic analysis. Extracted ITS reads were analyzed using default parameters of QIIME (Caporaso et al., 2010) with closed‐reference clustering for OTU picking with 97% identity and the UNITE database version 7.2 (Nilsson et al., 2019).

16S rRNA gene amplicon sequencing reads underwent taxonomic assignment using QIIME2 (Bolyen et al., 2019) which includes *vsearch dereplicate‐sequences* function for dereplication and *q2‐feature‐classifier* for taxonomic assignment and the SILVA v128 database (Quast et al., 2013) while default parameters of QIIME (Caporaso et al., 2010) with closed‐reference clustering for OTU picking with 97% identity and UNITE database version 7.2 (Nilsson et al., 2019) were used for ITS amplicon analysis.

#### 2.4.3 Metagenome assembly and reconstruction of individual genomes

MetaSPAdes v3.11.0 (Nurk, Meleshko, Korobeynikov, & Pevzner, 2017) was used with default parameters for assembly of WMS reads. Contig sequences were aligned to raw data using BWA (Li & Durbin, 2009) and placed into taxonomic bins with MetaBat2 (Kang, Froula, Egan, & Wang, 2015) using a minimum contig length of 2,000 bp. The comparison and dereplication of recovered genomes bins were completed using dRep (Olm, Brown, Brooks, & Banfield, 2017) with minimum primary Average Nucleotide Identity (ANI) 60% (Varghese et al., 2015) and minimum secondary ANI 95% (Jain, Rodriguez‐R, Phillippy, Konstantinidis, & Aluru, 2018). CheckM (Parks, Imelfort, Skennerton, Hugenholtz, & Tyson, 2015) was used to determine completeness and contamination of the best genomes selected by dRep analysis. Mash (Ondov et al., 2016) was used to cluster each best genome bin with its closest reference genome within RefSeq database and the dnadiff tool (Kurtz et al., 2004) was used to check their quality and distance to the clustered reference genomes. QUAST (Gurevich, Saveliev, Vyahhi, & Tesler, 2013) was employed to visualize genome bin contigs against the closest reference genome. Since it was not possible to assess the quality of fungal genomes with CheckM and dRep, all generated genome bins were also investigated for the identification of fungal genome bins. These bins were clustered against all the complete bacterial and fungal genomes in the RefSeq release 88 (O'Leary et al., 2015) using Mash and best fungal genome bin was selected through QUAST comparisons to the closest reference genome.

#### 2.4.4 *K. rhaeticus* genomic features and plasmid metagenome analysis

Comparing contig sequences against closest bacterial reference genomes revealed the presence of gapped regions (Figure 4). In order to understand the characteristics of these regions, the presence of repeats, rRNA loci, and plasmid sequences in the *K. rhaeticus* reference genome were determined using Tandem Repeats Finder (Benson, 1999), BLAST (Altschul, Gish, Miller, Myers, & Lipman, 1990), and PlasFlow (Krawczyk, Lipinski, & Dziembowski, 2018), respectively.

Plasmid contigs in the metagenome assembly results were also investigated using PlasFlow. The contigs identified as being derived from plasmids were extracted and analyzed separately. In order to investigate their taxonomic profile, all plasmid contigs were mapped against the NCBI Plasmid Genome Database (http://ftp://ftp.ncbi.nlm.nih.gov/genomes/archive/old_refseq/Plasmids/). Significant matches were defined using an alignment criteria of 95% sequence identity and minimum hit length of 90 bp (Zhang, Zhang, & Ye, 2011).

#### 2.4.5 Gene prediction and functional annotation

The gene prediction of three recovered *Komagataeibacter* genomes and plasmid metagenome were performed using Prokka version 1.4.0 (Seemann, 2014) while WebAUGUSTUS, a tool for eukaryotic gene prediction (Hoff & Stanke, 2013) was used for the recovered *Z. bailii* genome. GhostKOALA (Kanehisa, Sato, & Morishima, 2016) and Genome Properties (Haft et al., 2013; Richardson et al., 2019) were used for the functional characterization and the determination of complete pathways in both dominant genomes and plasmid metagenomes. Secondary metabolism genes were predicted and annotated using antiSMASH web server (Blin et al., 2017).

## 3 RESULTS AND DISCUSSION

### 3.1 Overview of sequencing results

WMS produced 2.3 billion reads in total, while amplicon sequencing yielded 1.85 million 16S rRNA gene reads and 1.80 million ITS1 reads. The number of total reads and retained reads following the quality control steps for each sample are shown in S1 File. On average, 13% of WMS reads, 5% of 16S rRNA gene reads, and 2% of ITS1 reads were removed from raw data based on quality control criteria of MGnify version 4.1.

### 3.2 Microbial composition of Kombucha samples

**Figure 2:**
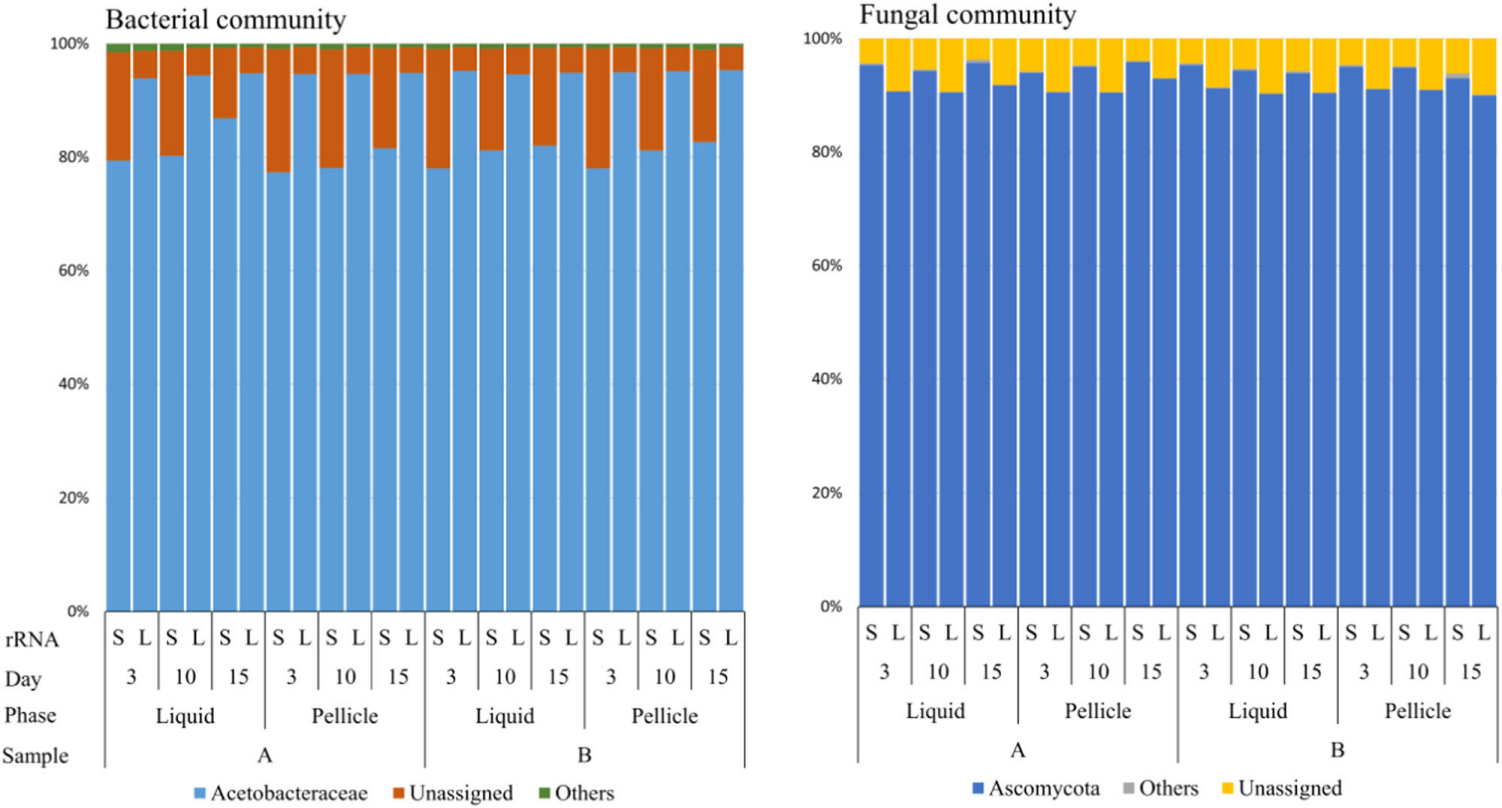
Relative abundances of bacterial families and fungal phyla in Kombucha samples using rRNA gene‐based (S: SSU, L: LSU) taxonomic analysis of WMS data. (The families and phyla accounting for a minimum of 1% of reads in at least one sample are displayed.)

The rRNA gene sequences were extracted from WMS data and used for the taxonomic analysis of Kombucha samples. rRNA gene‐based results showed that there are eight different bacterial phyla (minimum of 50 reads in at least one sample) in the two Kombucha samples: *Acidobacteria, Actinobacteria, Armatimonadetes, Bacteroidetes, Deinococcus‐Thermus, Firmicutes, Proteobacteria*, and *Verrucomicrobia*. Among these phyla, *Proteobacteria* was dominant (>99%) in all pellicle and liquid phase samples throughout the fermentation process (Figure 2). Since rRNA gene‐based taxonomic analysis of bacterial community did not allow genus level resolution, taxonomic assignments were restricted to the family level. On average, 82% of rRNA gene reads could be assigned at the family level, which revealed that 99% of the sequences assigned to the *Proteobacteria* phylum belong to the *Acetobacteraceae* family.

rRNA gene‐based taxonomic analyses of fungal community showed that *Ascomycota* (>80%) was the dominant phylum in both Kombucha samples (Figure 2). In addition, ITS1 sequences extracted from WMS raw data were assigned to the *Zygosaccharomyces* genus (>99%) across all samples.

Since WMS allows determination of both bacterial and fungal diversity simultaneously, it was possible to track the bacteria/fungi ratio. While the ratio increased over the 15‐day fermentation across all samples and phases, the 10th day samples showed great variability (Table 1). Other studies have reported that the number of bacterial and yeast cells increase until day 6 TO 10 of Kombucha fermentation and then start to decrease due to the lack of nutrients and increase in acidity of the environment (Chen & Liu, 2000; Coton et al., 2017; Teoh, Heard, & Cox, 2004). Thus, depending on when this change begins in individual Kombucha fermentations, the microbial composition can vary greatly between samples harvested at the same time.

**Table 1:** The relative abundance of bacteria, fungi, and bacteria/fungi ratio in Kombucha samples by WMS

Sample	Phase	Day	rRNA	Bacteria (%)	Fungi (%)	Bacteria/fungi
A	Liquid	3	SSU	95.13	4.10	23.20
			LSU	95.23	3.76	25.33
		10	SSU	25.93	68.32	0.38
			LSU	27.08	61.33	0.44
		15*	SSU	98.33	1.08	91.05
			LSU	98.65	1.04	94.86
	Pellicle	3	SSU	98.63	1.08	91.32
			LSU	98.62	1.13	87.27
		10	SSU	88.09	9.90	8.90
			LSU	88.09	9.90	8.90
		15	SSU	99.68	0.23	433.39
			LSU	99.68	0.23	433.39
B	Liquid	3	SSU	95.82	3.61	26.54
			LSU	95.99	3.39	28.32
		10	SSU	87.,31	11.36	7.69
			LSU	88.17	9.91	8.90
		15	SSU	97.80	1.59	61.51
			LSU	98.21	1.46	67.27
	Pellicle	3	SSU	96.57	2.93	32.96
			LSU	96.93	2.59	37.42
		10	SSU	97.66	1.83	53.37
			LSU	98.04	1.59	61.66
		15	SSU	98.72	0.26	379.69
			LSU	99.64	0.23	433.22

* Decontaminated sample: AS15.

**Figure 3:**
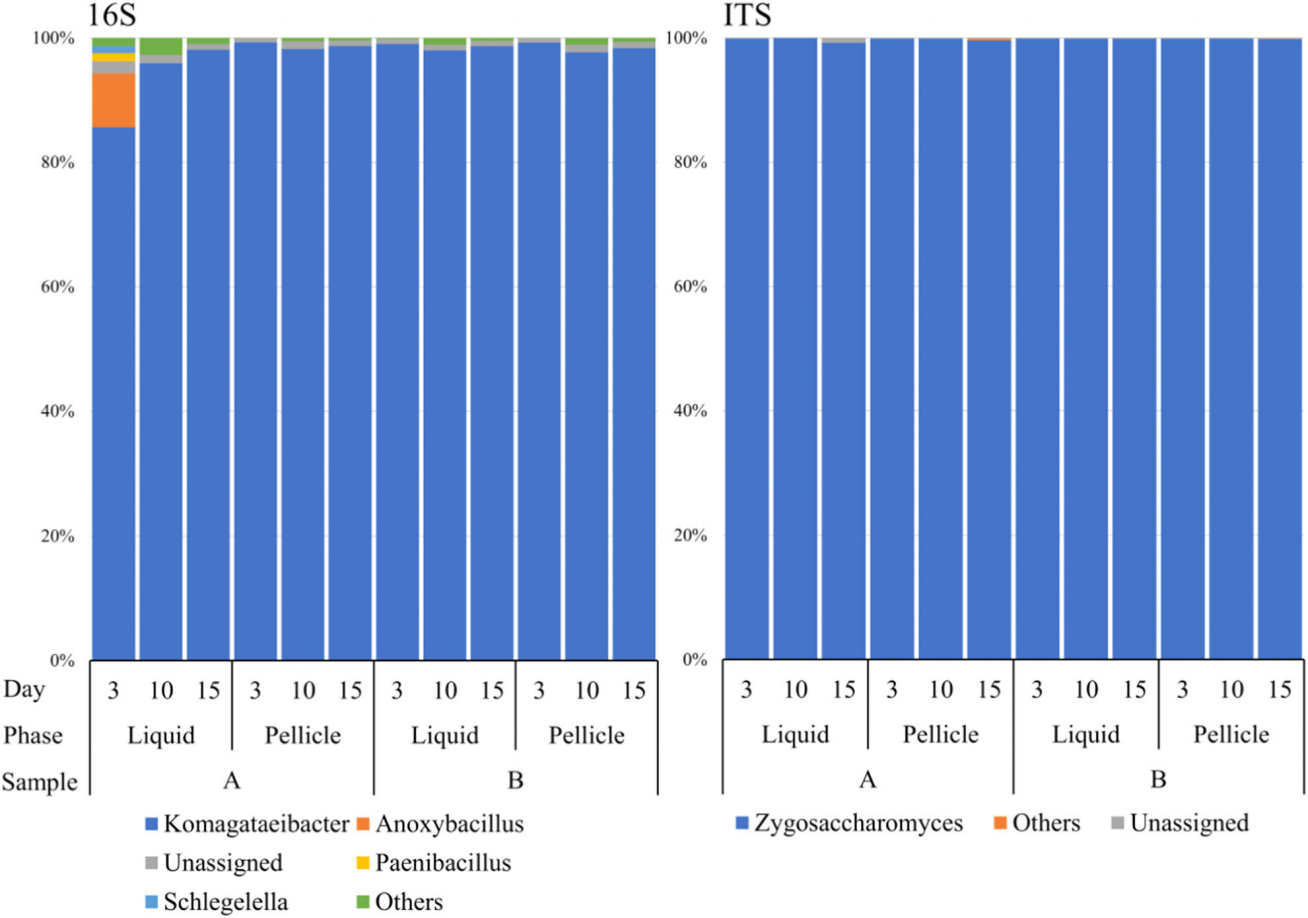
Relative abundances of bacterial and fungal genera in Kombucha samples using 16S rRNA gene and ITS1 amplicon sequencing. (The genera accounting for a minimum of 1% of reads in at least one sample are displayed.)

Amplicon‐based (16S rRNA gene and ITS1) NGS approaches were also applied and the taxonomic assignment results were compared to WMS (Figure 3). The 16S rRNA gene sequencing results showed strong agreement with WMS, except for AS3 in which the relative abundances of *Bacillaceae*, *Comamonadaceae*, and *Paenibacillaceae* families were 8.4%, 1.3%, and 1.2%, respectively in the 16S rRNA analysis. However, although lower in percentage terms, the relative abundance distribution patterns of these families in WMS samples were in line with 16S rRNA gene amplicon sequencing results. Thus, the differences between the two methods may result from bias in the 16S rRNA gene amplification step (Tremblay et al., 2015). Meanwhile, amplicon analyses showed that 99% of the sequences assigned to the *Proteobacteria* phylum belong to *Komagataeibacter* genus (relative abundance max: 99.3%, min: 85.6%) of the *Acetobacteraceae* family. ITS1 targeted amplicon analysis revealed that genus *Zygosaccharomyces* dominates the fungal community (>99%) across all samples, which was also consistent with WMS results (Figure 3).

The taxonomic and functional analyses of two Kombucha samples have shown that these two samples have same microbial communities. Comparing to Kombucha samples analyzed in other studies, the Turkish Kombucha samples have relatively lower microbial diversity in the sampling days which could be caused by previous long periods of fermentation or DNA isolation method. When we compared our taxonomic analysis results with previously reported NGS‐based Kombucha studies (Chakravorty et al., 2016; Coton et al., 2017; De Filippis et al., 2018; Marsh et al., 2014; Reva et al., 2015), we observed a high concordance with Marsh et al. (2014) which applied a very similar DNA isolation method and found ≥90% *Gluconacetobacter*, which formerly included *Komagataeibacter* (Yamada et al., 2012), and ≥95% *Zygosaccharomyces* at day 3 and day 10. This consistency suggests that DNA isolation protocol may have an effect on the detected DNA profiles that probably mirror microbiome composition.

### 3.3 Reconstruction of individual dominant genomes

In order to get a detailed insight into the higher order pathways, systems and functionality of Kombucha microbiome, WMS reads were assembled. The metagenome assembly statistics for Kombucha samples are shown in Table 2.

**Table 2:** General assembly and mapping statistics for Kombucha samples

Sample	No. of contigs	N50	N75	Largest contig (bp)	Singletons (%)
AS3	13,270	21,715	3,272	341,876	0.53
AS10	12,529	19,158	1,699	593,484	1.91
AS15	14,928	2,128	955	123,490	0.79
AP3	5,823	8,451	2,593	247,807	0.50
AP10	5,445	53,655	14,880	642,758	0.75
AP15	2,182	37,605	12,276	165,106	0.80
BS3	2,239	61,103	22,688	357,906	0.36
BS10	5,262	55,201	11,859	389,764	1.60
BS15	6,441	7,396	2,977	214,605	1.11
BP3	1,414	63,313	24,581	367,652	0.79
BP10	13,439	6,229	1,331	177,358	0.50
BP15	2,500	19,729	6,815	150,955	0.35

The average percentage of singleton reads in the metagenome assembly process was 0.83% while the average N50 value was 29,640. Taxonomic assignment and the quality assessment results for the bins are presented in S3 File. After dereplication, seven bins were obtained and used for further analyses. The quality assessment and taxonomic assignment results of these bins are presented in Table 3.

**Table 3:** Taxonomic assignment and the quality assessment results for the dereplicated genome bins, estimated by CheckM

Bin	Completeness (%)	Contamination (%)	Taxonomy
AS3_bin2	95.83	0	Bacteria
AS3_bin3	96.50	0	*Gluconacetobacter*
AS3_bin5	91.88	1.71	*Acidobacteriales*
AS3_bin13	100	0.93	*Rubrivivax*
AS3_bin14	82.66	1.01	*Lysobacter*
AS10_bin7	97.16	0.27	*Gluconacetobacter*
AS10_bin8	97.91	0.66	*Gluconacetobacter*

The taxonomic analysis results of dereplicated high quality bins showed that three of dereplicated bins belong to the *Gluconacetobacter* genus and are thus likely to be different species. Sample AS3 includes four dereplicated high quality bins classified as *Rubrivivax*, *Acidobacteriales, Lysobacter*, and Bacteria. rRNA gene‐based taxonomic assignments from WMS data showed that sample AS3 has the rRNA gene reads that could be classified at phylum level as *Proteobacteria*, which includes *Lysobacter* and *Rubrivivax*. However, there were no reads assigned to the Acidobacteria phylum, which includes *Acidobacteriales*. The discrepancy in the taxonomic assignment of these bins and rRNA‐based taxonomic analysis may result from the low resolution in rRNA‐based taxonomic analysis results from WMS.

**Figure 4:**
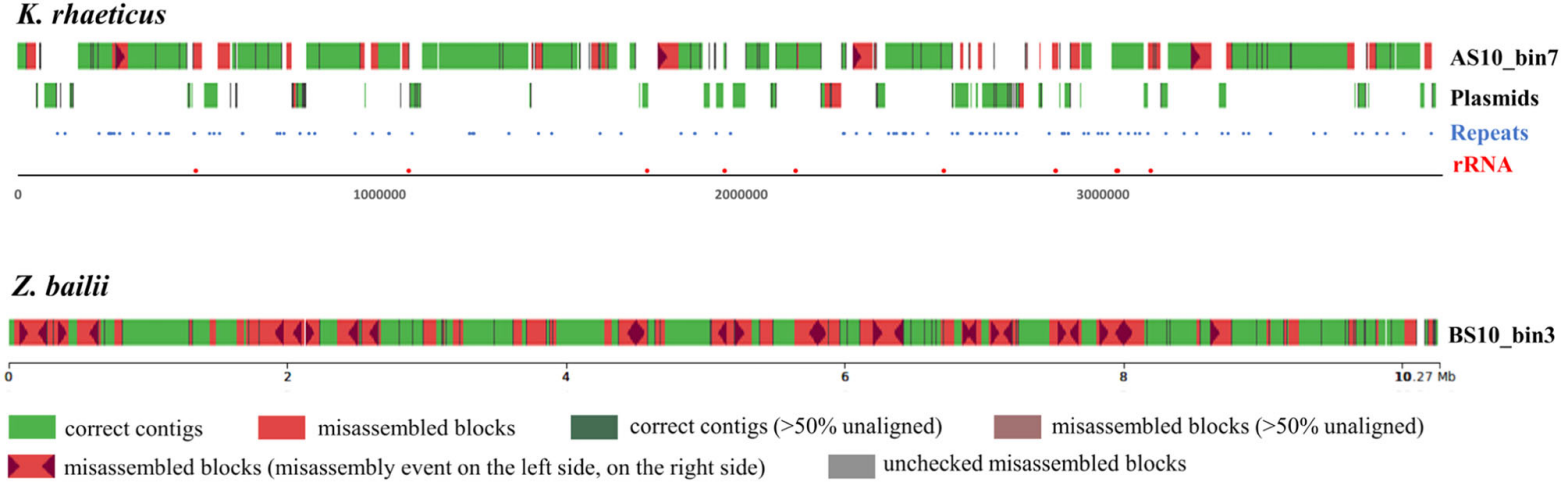
The distribution of contig sequences of *K. rhaeticus* and *Z. bailii* bins along reference genomes of *K. rhaeticus* AF1 and *Z. bailii* CLIB 213T.

None of the bins were classified as eukaryotic in the preliminary binning analysis results, which is to be expected as the panel of conserved genes used in CheckM for evaluation do not extend to eukaryotes. However, Mash and dnadiff analysis revealed that some bins cluster with the *Zygosaccharomyces bailii* genome (S4 File), and it was possible to reconstruct near complete genomes of the most abundant species from both bacterial and eukaryotic kingdoms. The clustering of the best genome bins of genome showed the presence of three *Komagataeibacter* genomes (one of which was classified as *K. rhaeticus* while the other two did not show species level identity with any *Komagataeibacter* genome), and one *Z. bailii* genome. The *K. rhaeticus* bin (AS3_bin7) showed 99.84% similarity with the isolate genome of *K. rhaeticus* AF1 from Kombucha (dos Santos et al., 2014) and *Z. bailii* bin showed 99.13% similarity with *Z. bailii* CLIB 213^T^ from wine (Galeote, Bigey, Devillers, Neuvéglise, & Dequin, 2013) and were visualized along the reference genomes using QUAST (Figure 4).

The *K. rhaeticus* is a cellulose producing species of *Komagataeibacter* genus and its presence in Kombucha has been previously reported (dos Santos et al., 2014; Semjonovs et al., 2017). *Komagataeibacter* species are usually found on various fruits and it is known that the cellulosic pellicle produced by these bacteria facilitates both adhesion to plants and protection against environmental factors such as UV (Williams & Cannon, 1989). As a member of the *Acetobacteraceae* family, *K. rhaeticus* also produces acetic acid to gain advantage over other competing microbial species in the environment (Yamada et al., 2012).

*Z. bailii* is a fermentative yeast with high tolerance to environmental stress conditions and is thus considered a food spoilage yeast by wine and beer producers (Kuanyshev, Adamo, Porro, & Branduardi, 2017; Steels, James, Bond, Roberts, & Stratford, 2002; Zuehlke, 2013). However, it has also been reported that this species has potential beneficial characteristics for fermented foods (Ciani, Comitini, Mannazzu, & Domizio, 2010; Jolly, Augustyn, & Pretorius, 2017; Kuanyshev et al., 2017; Xu, Zhi, Wu, Du, & Xu, 2017). Also, it is one of the most commonly reported yeast genera in Kombucha microbiome studies (Jayabalan et al., 2014; Marsh et al., 2014; Villarreal‐Soto, Beaufort, Bouajila, Souchard, & Taillandier, 2018). Despite the recent genomic‐based studies, the role of *Zygosaccharomyces* in fermented foods and its potential effects have not yet been elucidated in detail (Kuanyshev et al., 2017). Interestingly, application of purified cellulase from *Z. bailii* to the tea production processes has been shown to improve tea quality by release of aroma compounds (Murugesan, Angayarkanni, & Swaminathan, 2002), suggesting this fungi could be important for Kombucha flavor.

During Kombucha fermentation, *Z. bailii* metabolizes sucrose to produce glucose and fructose. *Z. bailii* preferentially metabolizes fructose to produce ethanol, even when glucose is present in the growth medium as a carbon source (Merico, Capitanio, Vigentini, Ranzi, & Compagno, 2003). *Komagataeibacter* species use glucose to produce gluconic acid, and ethanol to produce acetic acid, which increase the acidity of the environment and inhibit growth of other species except *Z. bailii*, which has a high tolerance to acetic acid and other weak acids and grows even faster in low acetic acid and lactic acid concentrations (Dang, Vermeulen, Ragaert, & Devlieghere, 2009). In addition, glucose and fructose are used by *Komagataeibacter* species in the production of a cellulosic pellicle, by which it moves to the top of the liquid phase to provide access to the oxygen and thus further stimulate cellulose production (Chang et al., 2001). These complimentary metabolic activities and cross talk of these dominant species create an environment that prevents the growth of other microorganisms.

### 3.4 *K. rhaeticus* genomic features and plasmid metagenome of Kombucha samples

A high fraction (98.89%) of the *Z. bailii* CLIB 213^T^ reference genome was covered at 95% identity threshold by deep sequencing and metagenome assembly. However, the matched fraction of the *K. rhaeticus* was only 69.87% at 95% identity threshold and the visualization of the binned contig sequences against *K. rhaeticus* AF1 genome revealed the presence of many gapped regions. In order to understand whether repetitive regions or rRNA genes cause these gaps, repeats and rRNA gene loci on the reference genome were determined (Figure 4). In addition, the *K. rhaeticus* AF1 reference genome was analyzed for plasmids, since it is known that current metagenomic binning approaches are not efficient in binning plasmids (Beaulaurier et al., 2018). Interestingly, these results demonstrated that the presence of plasmid scaffolds corresponded to the observed gap regions (Figure 4), indicative of possible misassembly in the reference genome. Removal of the plasmid scaffolds from *K. rhaeticus* AF1 reference genome increased the matched genome fraction by the metagenome assembled genome increased to 90.42%.

The plasmid contigs in the metagenome assembly results were also identified and extracted from assemblies for further analyses. For taxonomic assignments, in total, 11,337 plasmid contigs were mapped against NCBI Plasmid Genome Database including 6,113 plasmid genomes. 1204 (10.6%) plasmid contigs had a match in the database with hit lengths ranging from 90 to 24,104 bp. Note that 78.8% of the database matches belongs to *Gluconacetobacter* genus, 8.9% belongs to *Acetobacter*, and 3.6% belongs to *Gluconobacter* which shows that a high percentage of Kombucha plasmids originate from *Komagataeibacter* species.

### 3.5 Gene prediction and functional annotation

The assembled contig‐based approach was employed to explore the potential functional profile of the dominant genomes and plasmids. The full list of complete pathways of *Komagataeibacter* and *Z. bailii* genome bins are provided in the S5 File. Among the complete pathways identified in these recovered genomes, particular attention was given to those that may be involved in the interactions between the dominant species or have potential implications for the characteristics of the Kombucha fermentation.

Kombucha is characterized with the production of a cellulosic pellicle by bacteria during the fermentation process and it is well known that the structure of cellulose synthase operons can vary significantly even between the strains of the same species (Römling & Galperin, 2015). The structure of cellulose synthase operons in the recovered *Komagataeibacter* genomes was therefore assessed. It was found that two genomes to have four cellulose synthase operons, similar to previous studies (Liu et al., 2018), while one genome had three cellulose synthase operons.

The functional analyses also showed that the *K. rhaeticus* genome bin carrries complete pathways for the biosynthesis of vitamin B1, vitamin B7, vitamin B12 (S6 File) which were annotated for the first time in *K. rhaeticus*. The presence of B‐group vitamins in Kombucha have been reported previously (Jayabalan et al., 2014). It is also known that *Z. bailii* requires B‐group vitamins for its growth (Stratford & Capell, 2003). Thus, it is predicted that the vitamin biosynthesis by *Komagataeibacter* species and their use by *Z. bailii* may be an important interaction between these species in Kombucha.

The analysis of the recovered *Z. bailii* genome showed the presence of important pathways and genes such as GABA (gamma‐aminobutyrate) shunt which has an important role in oxidative stress tolerance in *S. cerevisiae* (Coleman, Fang, Rovinsky, Turano, & Moye‐Rowley, 2001). Although there are conflicting reports about the benefits of GABA as a food supplement (Boonstra et al., 2015), the production of GABA‐enriched fermented foods have been studied in many different research groups (Dhakal, Bajpai, & Baek, 2012).

To give a more complete picture in terms of encoded pathways and systems, Kombucha plasmid contigs were also analyzed. On average, 25% of all predicted plasmid genes could be annotated. The full list of complete plasmid pathways is provided in the S6 File. Notably, a complete Type IV secretion system (T4SS) which is responsible for conjugative transfer of plasmid DNA, release or uptake of DNA and translocation of effector macromolecules, was detected in plasmid metagenome.

### 3.6 Screening secondary metabolite gene clusters

Since the biosynthesis of secondary metabolites was one of the most enriched categories in both the dominant genomes and plasmid metagenomes, a search for the secondary metabolite gene clusters was performed using antiSMASH (S7 File). Bacteriocin gene clusters that show no similarity to any known gene cluster were detected in two *Komagataeibacter* genomes and plasmid metagenome, which implies the potential of these bacteria to use these antimicrobial peptides against other species in the environment. In addition, terpene gene clusters detected in all *Komagataeibacter* genomes, the *Z. bailii* genome, and plasmid metagenomes potentially contribute to the specific odor of Kombucha (Audrain, Farag, Ryu, & Ghigo, 2015).

The screening of plasmid metagenomes for secondary metabolite genes also indicated the presence of methanobactin genes, a family of copper‐binding peptides that might be responsible for the copper biosorption feature of Kombucha (Razmovski & Šćiban, 2008) and thus its toxicity when brewed in metal containers (Jayabalan et al., 2014). Interestingly, it was recently reported that the *Z. bailii* genome includes the ZbHAA1 gene that encodes a bifunctional transcription factor that controls both the acetic acid and copper stress response regulons which potentially provides high tolerance to these environmental stresses (Palma et al., 2017). These findings may present another important example of the mutually beneficial functional characteristics of the dominant species of Kombucha.

## 4 CONCLUSIONS

In this study, we determined the microbial composition of Kombucha using a combination of WMS and amplicon (16S rRNA gene and ITS1) sequencing. Taxonomic analyses results revealed a stable low diversity microbial ecosystem from day 3 to day 15. The microbial community was dominated by three *Komagataeibacter* species and *Z. bailii* across all samples and phases. The gene prediction and functional annotation of the reconstructed genomes of these dominant species and plasmid metagenomes have shown the presence of various potential functional properties, such as vitamin production, copper binding, tolerance to acidic pH, and production of antimicrobials. The findings of this study provide novel information on *Komagataeibacter* and *Zygosaccharomyces* species genomes and potential functional roles and interactions. In future studies, microbial dynamics and their effects on Kombucha characteristics could be explored more comprehensively by combining metagenomics and metabolomics approaches.

## CONFLICTS OF INTEREST

The authors declare that they have no conflicts of interest.

## DATA AVAILABILITY

Amplicon sequencing (16S rRNA gene and ITS1) and WMS reads produced in this study are available at the European Nucleotide Archive (ENA) under the accession numbers ERP104502 and ERP024546, respectively.
